# *In vitro* and *in vivo* evaluation of docetaxel-loaded stearic acid-modified *Bletilla striata* polysaccharide copolymer micelles

**DOI:** 10.1371/journal.pone.0173172

**Published:** 2017-03-23

**Authors:** Qingxiang Guan, Guangyuan Zhang, Dandan Sun, Yue Wang, Kun Liu, Miao Wang, Cheng Sun, Zhuo Zhang, Bingjin Li, Jiayin Lv

**Affiliations:** 1 School of Pharmacy, Jilin University, Changchun, China; 2 Faculty of Chemistry, Northeast Normal University, Changchun, China; 3 China-Japan Union Hospital of Jilin University, Changchun, China; 4 The Second Hospital of Jilin University, Changchun, China; Xi′an Jiaotong University, CHINA

## Abstract

*Bletilla striata* polysaccharides (BSPs) have been used in pharmaceutical and biomedical industry, the aim of the present study was to explore a BSPs amphiphilic derivative to overcome its application limit as poorly water-soluble drug carriers due to water-soluble polymers. Stearic acid (SA) was selected as a hydrophobic block to modify *B*. *striata* polysaccharides (SA-BSPs). Docetaxel (DTX)-loaded SA-BSPs (DTX-SA-BSPs) copolymer micelles were prepared and characterized. The DTX release percentage *in vitro* and DTX concentration *in vivo* was carried out by using high performance liquid chromatography. HepG2 and HeLa cells were subjected to MTT (3-(4, 5-dimethylthiazol-2-yl)-2, 5-diphenyl tetrazonium bromide) assay to evaluate the cell viability. *In vitro* evaluation of copolymer micelles showed higher drug encapsulation and loading capacity. The release percentage of DTX from DTX-SA-BSPs copolymer micelles and docetaxel injection was 66.93 ± 1.79% and 97.06 ± 1.56% in 2 days, respectively. The DTX-SA-BSPs copolymer micelles exhibited a sustained release of DTX. A 50% increase in growth inhibition was observed for HepG2 cells treated with DTX-SA-BSPs copolymer micelles as compared to those treated with docetaxel injection for 72 h. DTX-SA-BSPs copolymer micelles presented a similar growth inhibition effect on Hela cells. Furthermore, absolute bioavailability of DTX-SA-BSPs copolymer micelles was shown to be 1.39-fold higher than that of docetaxel injection. Therefore, SA-BSPs copolymer micelles may be used as potential biocompatible polymers for cancer chemotherapy.

## Introduction

Self-assembled copolymer micelles consisting of amphiphilic block copolymer in aqueous medium are receiving considerable attention as gene and drug nanocarriers because of their particular characteristics [1–3]. Micelles always have a unique core—shell backbone composed of a hydrophilic shell and a hydrophobic core [4]. Hydrophobic drugs can be incorporated into the hydrophobic core of copolymer micelles, whereas the hydrophilic shell can stabilize and protect the drug in the aqueous medium. Furthermore, the hydrophilic shell can prolong the blood circulation time of micelles as a result of steric stabilization, which helps micelles escape mononuclear phagocyte system uptake after intravenous administration[5, 6]. These self-assemblies have potential uses in medicine and biotechnology because of their unique core—shell backbone[7], various amphiphilic block copolymers have been synthesized and their characteristics have been investigated widely[8, 9]. Recently, many efforts have been performed to prepare non-toxic and biocompatibility amphiphilic block copolymers on the basis of natural polysaccharides[10].

The water soluble polysaccharides(dextran, pullulan and heparin) have been modified to obtain the amphiphilic polymers by including the hydrophobic groups, such as alkyl, aralkyl, and deoxycholic [11]. The amphiphilic polymers were liable to self-aggregated copolymer micelles because of inter and/or intra molecular hydrophobic interactions in water medium [12, 13]. The amphiphilic polymers have been used with various targeting ligands, such as antibodies, peptides and anti-cancer drugs, greatly improving the precision of drug targeting to the tumor cells via endocytosis mechanisms [14, 15].

*Bletilla striata (Thunb*.*) Reichb*.*f*. polysaccharides (BSPs), the major active ingredients of *B*. *striata*, were extracted from the tubers, and are composed of (1→2)-α-D-mannopyranose and (1→4)-β-D-glucose, as characterized by nuclear magnetic resonance spectroscopy [16]. BSPs have been used in pharmaceutical and biomedical industry because of their negligible cytotoxic effects and properties, such as biocompatibility and biodegradability [17–19]. Therefore, BSPs may be potential candidates for various pharmaceutical applications, such as drug delivery system [20]. The BSPs have shown to inhibit the hepatocellular carcinoma growth after transarterial chemoembolization [21]. The 5-fluorouracil *B*. *striata* microspheres were characterized with long-term high efficacy and low toxicity compared to 5-fluorouracil injection [22]. However, BSPs are water-soluble polymers which limit its use as a poorly water-soluble drug carrier. To solve this problem, alkyl, aralkyl, and deoxycholic acid were used to modify water-soluble copolymer to improve its hydrophobic property [12].

**Fig 1:**
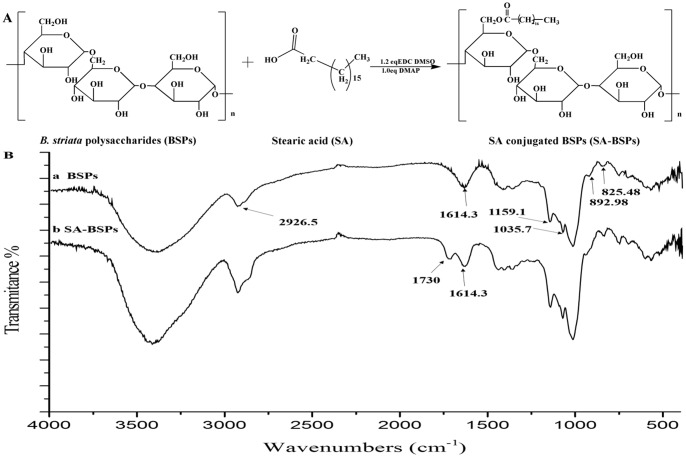
Synthetic route of SA-BSPs copolymer and Fourier Transform Infrared (FTIR) spectra. (A) Stearic acid (SA) conjugated B. striata polysaccharides (BSPs) was synthetized by grafting SA onto the hydroxyl group of BSPs. (B) Figure showing FTIR spectra of BSPs (a) and SA-BSPs (b). FTIR experiments were recorded at 25°C on Shimadzu 8300 FTIR spectrometer with a KBr tablet with the range of 400–4000 cm^-1^ (Shimadzu).

In the present study, stearic acid-modified *B*. *striata* polysaccharide (SA-BSPs) copolymers were synthesized by covalent attachment of stearic acid to polysaccharides and amphiphilic polymers with possible application in pharmaceutical industry were obtained. The chemical structure of the SA-BSPs is presented in Fig 1A. The SA-BSPs were characterized by using Fourier transform infrared (FTIR) spectroscopy, ^1^H nuclear magnetic resonance (^1^H-NMR) spectroscopy, and critical aggregation concentrations (CAC) techniques. Docetaxel was selected for the preparation of DTX-SA-BSPs copolymer micelles. Polydispersity index, particle diameter, *zeta* potential, drug loading capacity (LC), encapsulation efficiency (EE), drug release *in vitro* and absolute bioavailability *in vivo* were also presented. Further, the antitumor activity of DTX-SA-BSPs and SA-BSPs copolymer micelles was measured *in vitro* using Hela human cervical cancer cells and HepG2 human liver cancer cells.

## Materials and methods

### Materials

Docetaxel injection (Duopafei^®^) was purchased from Qi Lu Pharmaceutical Co., Ltd. (Jinan, China). Acetonitrile and methanol were supplied by Fisher (USA, chromatographic grade). Docetaxel was provided by Shanghai Boyle chemical Co., Ltd (Shanghai, China). *Bletilla striata* polysaccharides were purchased from Shanxi Pioneer Biotech Co., Ltd (Shanxi, China). 4-Dimethylaminopyridine (DMAP) and 1-ethyl-3-[3-(dimethyl amino) propyl] carbodiimide (EDC) were supplied by Energy Chemical Co., Ltd. (Shanghai, China). Stearic acid (SA) was purchased from Sino pharm Chemical Regent Co., Ltd (Beijing, China). All the other reagents used were of analytical purity grade and obtained commercially.

### Synthesis of SA-BSPs copolymer

The SA-BSPs copolymers were synthesized using SA, EDC, and DMAP, as shown in Fig 1A. SA (1.812 g), EDC (1.380 g), and DMAP (0.7636 g) were added in 15 mL dimethyl sulfoxide (DMSO) solution and then the mixture was stirred for 2 h at 25°C. The BSPs (5.774 g) were dissolved in 20 mL of DMSO under stirring condition. The BSPs solution was then added dropwise to the mixed solution (15 mL) at 25°C and kept for 48 h at 38°C, as described [23]. The reaction solution was diluted 10-fold with cold ethanol. The precipitate was recovered by filtration, washed three times, first with ethanol (100 mL) and then with diethyl ether (100 mL), and dried in vacuum at 50°C.

### Characterization of SA-BSPs copolymers

The BSPs and SA-BSPs copolymers were analyzed using FTIR spectroscopy on a Shimadzu 8300 FTIR spectrometer with a KBr tablet with the range of 400–4000 cm^-1^ (Shimadzu, Tokyo, Japan).

The ^1^H NMR spectra of the samples (5 mg) were determined in DMSO-d_6_ solution (500 μL) using a 500 MHz NMR spectrometer (AVIII, Bruker, 500 MHz) at 25°C, as described [24, 25]. All the spectra were analyzed and processed with Bruker Topspin version 3.0 software. The substituted degree (DS) of the SA-BSPs group was measured by ^l^H NMR.

DS was calculated according to the following equation described in [21]:

$$DS\;\%=\left(A_{\delta 1.24}/32+A_{\delta 0.85/3}\right)/\left(A_{\delta 5.53}+A_{\delta 4.55}\right)*100\;\%$$

A_δ1.24_ was the peak area of methylene protons and A_δ0.85_ was the peak area of methyl protons. A_δ5.43_ was the peak area of hydrogen [H (1, 6)] protons and A_δ4.55_ was the peak area of hydrogen [H (1, 4)] protons.

The self-aggregation property of SA-BSPs was measured by RF-5301 fluorescence spectrophotometer with pyrene as a hydrophobic fluorescence probe [26]. The initial concentration of pyrene solution was 1.2×10^−6^ mol/L (M). The pyrene solution was mixed with SA-BSPs copolymer micelles solution to obtain a SA-BSPs with concentration range of 1.0×10^−5^~1 mg/mL, and the final pyrene concentration was 6.0×10^−7^ M. Fluorescence emission spectra was investigated by excitation at the wavelength of λ ex = 334 nm. Slit width was set at 5 nm and 2.5 nm for the excitation and emission respectively. Based on the pyrene excitation spectra and red shift of the spectra with SA-BSPs concentration increase, the critical aggregation concentrations of the SA-BSPs self-aggregates were calculated by the Benesi-Hildebrand relationship [27].

### Preparation of DTX-SA-BSPs copolymer micelles

The SA-BSPs (50 mg) dissolved in 4 mL DMSO solution was transferred into a cellophane membrane dialysis bag and dialyzed with 500 mL of deionized water each time for 7 times [25]. Deionized water (500 mL) was changed every 2 h for 4 times and then every 8 h for 3 times under stirring condition at 100 rpm/min at 25°C. The copolymer micelles solution filtered through a 0.45 μm membrane filter was adjusted to 100 mL by adding deionized water. Docetaxel (20 mg) was completely dissolved in 10 mL absolute ethanol and then slowly added into copolymer micelles solution dropwise under magnetic stirring condition at a speed of 100 rpm/min for 2 h. The DTX-SA-BSPs copolymer micelles were harvested by evaporating the ethanol with vacuum rotary evaporation instrument and the volume was adjusted to 100 mL by adding deionized water. The DTX-SA-BSPs copolymer micelles with 2% mannitol (W/V) as lyoprotectant were placed into glass dishes, frozen for 2 h under -20°C and then placed in a freeze-dryer (Free Zone 12 Liter, Labconco Corporation manufactures laboratory equipment Co., USA) at -40°C for 48 h with a pressure of 50 Pascal to get the lyophilized DTX-SA-BSPs copolymer micelles.

### Characterization of DTX-SA-BSPs copolymer micelles

The *zeta* potential and particle diameter of DTX-SA-BSPs copolymer micelles were measured using a dynamic light scattering (DLS) particle size analyzer with a scattering angle of 90° (Zetasizer Nano ZS, Malvern Instruments, UK) at 25°C, as described [28]. All the experiments were carried out in triplicate and data were expressed as mean values with their standard deviations (S.D.).

The surface morphology of the DTX-SA-BSPs copolymer micelles was determined using the transmission electron microscope (TEM, JEM-2010, Japan). Each sample was prepared by the same procedure as described for *zeta* potential measurements [29]. A droplet from each sample was stained with 1% phosphotungstic acid solution for 10 min and was placed on a copper grid. Subsequently, the excess solution was removed with filter paper. The sample was dried at 25°C, and then was subjected to TEM observation. All the experiments were carried out in triplicate.

For measuring the drug content and loading efficiency, the DTX in DTX-SA-BSPs copolymer micelles was separated from the copolymer micelles by centrifugation at a speed of 12,000 rpm for 10 min. The clear supernatant was analyzed for the contents of DTX by high performance liquid chromatography (HPLC) (LC-20AT, Shimadzu) at 230 nm. The drug LC and EE of DTX-SA-BSPs copolymer micelles were calculated as follows:

LC%=Weight of DTX in the SA-BSPs/Weight of SA-BSPs*100 %

EE%=Weight of DTX in the SA-BSPs/Weight of the feeding DTX*100 %

### *In vitro* study

#### *In vitro* drug release

The *in vitro* release profile of DTX from DTX-SA-BSPs copolymer micelles was investigated by using the dialysis method. The DTX-SA-BSPs copolymer micelles and docetaxel injection were suspended in 3 mL of distilled water, bringing the final concentration of DTX to 100 μg/mL, and the solution was transferred into a cellophane membrane dialysis bag (8–12 kDa). The dialysis bag was then suspended in 15 mL phosphate buffer saline (PBS, pH 7.4) with 0.2% of Tween 80, and subjected to horizontal stirring at a speed of 100 rpm/min at 37±0.5°C [30]. An aliquot of 5 mL sample was withdrawn at different time points (0, 1, 2, 3, 5, 7, 8, 9, 24 and 48 h) and the solution was compensated with an equal volume of the fresh medium maintained at same temperature. The content of DTX was measured by using HPLC. Sink condition was maintained throughout the release periods. All the experiments were performed in triplicate.

#### *In vitro* cytotoxicity study

The *in vitro* cytotoxicity was carried out on Hela and HepG2 cells using the MTT (3-(4, 5-dimethylthiazol-2-yl)-2, 5-diphenyl tetrazonium bromide) assay, as described [31]. Briefly, Hela and HepG2 cells with 5×10^4^ viable cells per well of initial density were plated in a 96-well plate and incubated for 24 h. The cells were exposed to different doses of docetaxel injection, blank SA-BSPs copolymer micelles and DTX-SA-BSPs copolymer micelles at 37°C. The DTX concentrations of 0.0005, 0.005, 0.05, and 0.5 μg/mL were used. After 72-h incubation, 20 μL of MTT solution (5 mg/mL) was added to each well of the plate. Following 4 h of MTT treatment, 150 μL of DMSO was added to each well to dissolve the formazan crystals, and the absorbance was measured at 492 nm using a microplate reader (FL600, Bio-Tek Inc., Winooski, VT). The cell viability (represented in %) was calculated according to the following equation [26]

$$Cell\;viability\;\%=\left(OD_{492,\;sample}-OD_{492,\;blank}\right)/\left(OD_{492,\;control}-OD_{492,\;blank}\right)*100\;\%$$

OD_492, sample_ represents the values obtained from the samples treated with docetaxel injection, blank SA-BSPs copolymer micelles or DTX-SA-BSPs copolymer micelles; OD_492, control_ represents the values obtained from the cells treated with incubated solution and OD_492, blank_ represents the values obtained from only incubated solution.

### *In vivo* study

The *in vivo* bioavailability assay of DTX-SA-BSPs copolymer micelles was conducted according to the Guidelines for Care and Use of Laboratory Animals. Male Wistar rats were obtained from the Laboratory Animal Center of Jilin University (Changchun, China). The rats were maintained at 20 ± 2°C and 50–60% relative humidity. A 12-h dark/light cycle was maintained throughout the study. All the animals were maintained in fasting condition for 12 h with free access to water before the experiment. The animals were randomly divided into two groups, and each group received either DTX-SA-BSPs copolymer micelles or docetaxel injection at an equivalent dose of 20 mg/kg (DTX/rat body weight) through the tail vein injection. Blood sample (0.5 mL) was collected in heparinized tubes from the retro-orbital plexus while the rats were anesthetized under diethyl ether inhalation after 0, 10, 30, 60, 120, 180, 240, 300, and 360 min of vein injection. The blood sample was then separated from the heparinized blood by centrifugation using a centrifuge 5415C (Eppendorf, Germany), and stored at -20°C until analysis [28]. All the data were analyzed using the DAS 2.1 pharmacokinetic software (a program by Chinese Pharmacological Society, China), and the results were represented as mean values with standard deviations. The study was conducted in accordance with the Guide for the Care and Use of Laboratory Animals published by the National Institutes of Health and with the recommendations and approval of the Ethics Committee on Animal Experiments of Jilin University. All efforts were made to minimize suffering. All rats were killed by barbiturate overdose after experiments.

## Results

### Characterization of SA-BSPs copolymers and DTX-SA-BSPs copolymer micelles

**Fig 2:**
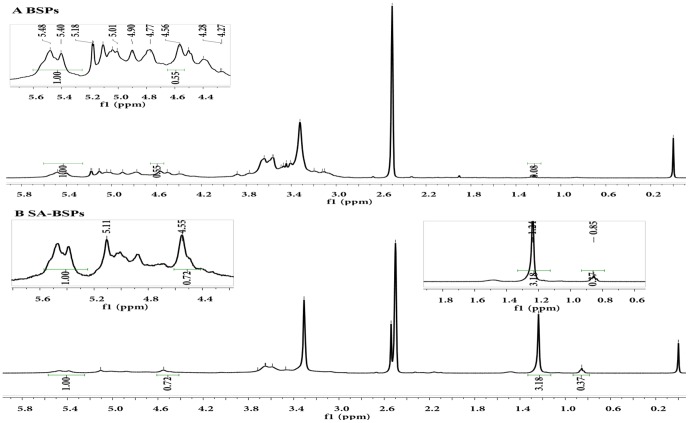
^1^H nuclear magnetic resonance (^1^H-NMR) spectra of BSPs (A) and SA-BSPs (B) in DMSO_d6_. ^1^H-NMR spectra indicated the generation of methylene (δ_1.24_) and methyl (δ_0.85_) protons after the addition of SA to the reaction mixture containing BSPs.

Synthetic route of SA conjugated BSPs is presented in Fig 1A. SA, EDC, and DMAP were used to synthesize the SA-BSPs amphiphilic copolymer. The FTIR spectra of BSPs and SA-BSPs are shown in Fig 1B. Compared to the standard spectrum of BSPs, a new peak showing the characteristic absorption at 1730 cm^-1^ was observed for SA-BSPs. The characteristic absorption peaks at 892.98 and 825.48 cm^-1^ showed the existence of ß-glucosyl and mannose residues, respectively. The absorption peaks at 1035.7 and 1159.1 cm^-1^ indicated pyran-glycosylation of BSPs. The presence of methyl (-CH_3_) group is indicated by a strong absorption at 2926.5 cm^-1^. Characteristic peak at 3388.55 cm^-1^ can be attributed to the hydroxyl group (-OH) stretching. Fig 2 show the ^1^H NMR spectra of BSPs and SA-BSPs in DMSO_*-d6*_, and the peak areas of ^1^H NMR signals are listed in Table 1. The δ_1.24_ and δ_0.85_ ppm correspond to the peak of methylene and methyl protons, respectively. Hydroxyl proton signals were observed at δ_4.5–5.6_ ppm. The δ_5.43_ ppm peak corresponds to (1→6)-linked hydrogen protons, while δ_4.55_ ppm peak indicated the presence of (1→4)-linked hydrogen protons in BSPs. Furthermore, the DS of SA-BSPs was 12.94%, which was calculated from the peak areas (Table 1) of ^1^H NMR signals.

**Table 1:** Peak area of δ_1.24_, δ_0.85_, δ_5.43_ and δ_4.55_^1^H NMR signals.

Sample	A_δ5.43_	A_δ4.55_	A_δ1.24_	A_δ0.85_
SA-BSPs	0.94	0.64	2.85	0.33

**Fig 3:**
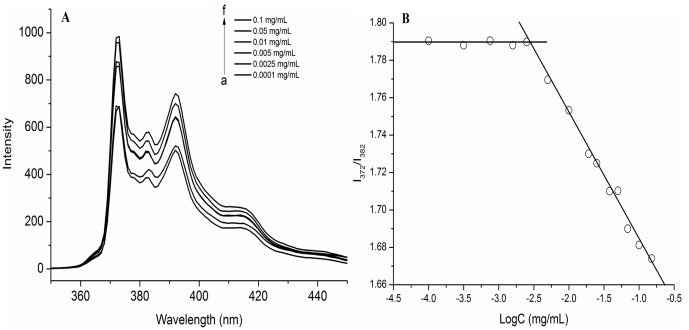
Fluorescence emission spectra of SA-BSPs copolymer micelles in distilled water at 25°C. (A) Emission spectra of pyrene (6×10^−7^ M) at the presence of SA conjugated BSPs. (B) Plot of the intensity ratio of I_372_/I_382_ from emission spectra *vs* log C of the SA-BSPs copolymer micelles.

Critical aggregation concentration (CAC) spectra are shown in Fig 3. The fluorescence intensity of SA-BSPs copolymer micelles was shown to increase significantly with an increase in the concentration (Fig 3A). A clear cross point (Fig 3B) was obtained for changes in I_372_/I_382_, and the CAC was approximately 3.09 μg/mL.

**Fig 4:**
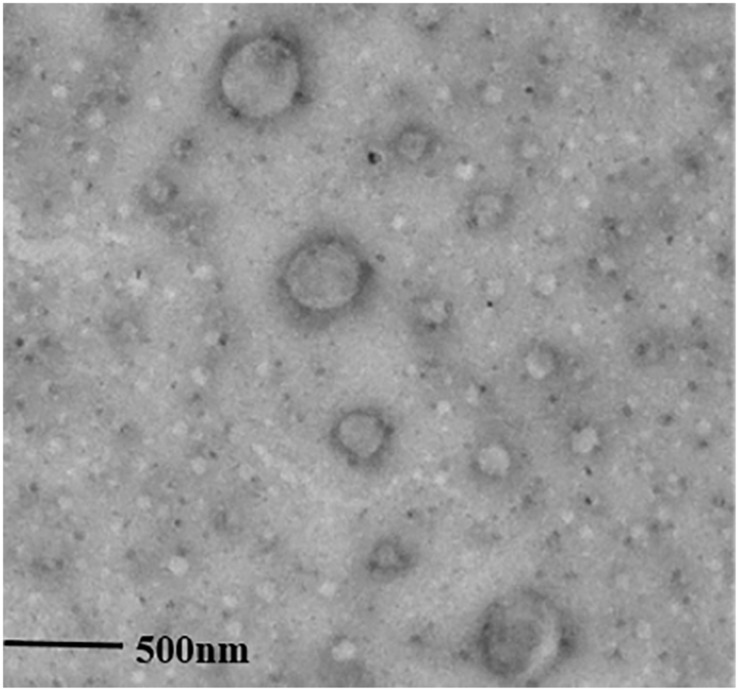
Transmission electron microscopy image of DTX-SA-BSPs copolymer micelles. The TEM image was offered with a magnitude 20000× and scale 500 nm.

The average diameter, *zeta* potential, EE % and LC % of the copolymer micelles are shown in Table 2. The average particle diameter of SA-BSPs copolymer micelle was 60.52 ± 3.34 nm, whereas the *zeta* potential was -20.12 ± 0.57 mV. The study showed that the LC and EE percentages were improved with the increase of drug *verse* carrier mass ratio from 1:20 to 1:9 (W/W). The EE and LC observed were 81.11 ± 0.18% and 9.13 ± 0.17%, respectively, when the drug *versus* carrier mass ratio was 1:9. The values of LC and EE decreased when the mass ratio of the drug *versus* carrier was beyond 1:8. The spherical morphology of DTX-SA-BSPs copolymer micelles is shown in Fig 4.

**Table 2:** Characterization of DTX-SA-BSPs copolymer micelles.

Drug/ carrier (W/W)	EE (%)	LC (%)	Average diameter (nm)	*Zeta* potential (mV)
0:9	—	—	60.52±3.34	-20.12±0.57
1:20	87.45±0.12	4.39±0.11	86.37±5.01	-20.37±0.25
1:10	84.16±0.23	8.46±0.14	98.97±4.80	-21.22±0.19
1:9	81.11±0.18	9.13±0.17	97.01±3.17	-19.56±0.22
1:8	69.74±0.22	8.75±0.13	90.56±3.90	-20.36±0.57

#### *In vitro* drug release

**Fig 5:**
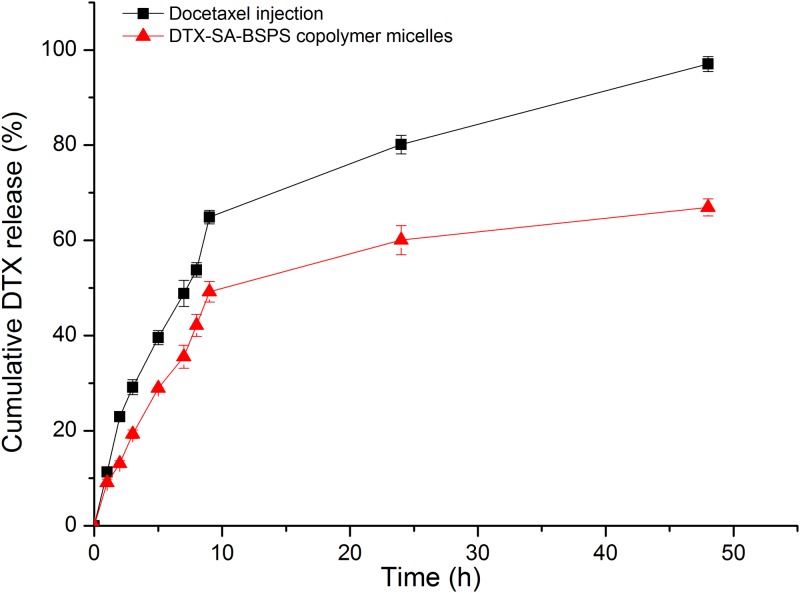
*In vitro* release profiles of DTX from docetaxel injection (-■-) and DTX-SA-BSPs copolymer micelles (-▲-) in pH 7.4 phosphate-buffered saline containing 0.2% of Tween 80 at 37 ± 0.5°C.

The release profiles of DTX from DTX-SA-BSPs copolymer micelles and docetaxel injection are shown in Fig 5. The release percentage of DTX from docetaxel injection was faster and higher 64.87 ± 1.44% than that from DTX-SA-BSPs copolymer micelles 49.21 ± 2.15% in the phosphate buffer saline (pH 7.4) solution containing 0.2% of Tween 80 at 9 h. The DTX-SA-BSPs copolymer micelles tended to be stable even after 10 h. The release percentage of DTX from DTX-SA-BSPs copolymer micelles was 60.04 ± 3.06% in the first 24 h and 66.93 ± 1.79% in 2 days. The release percentage of DTX from docetaxel injection was approximately 100% after 48 h.

#### *In vitro* cytotoxicity study

**Fig 6:**
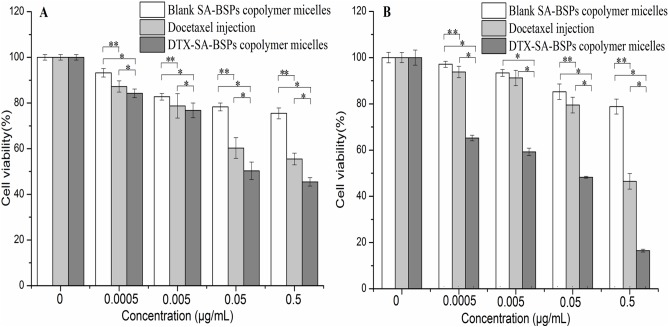
Cytotoxic effects of SA-BSPs copolymer micelles, docetaxel injection and DTX-SA-BSPs copolymer micelles on Hela (A) and HepG2 (B) cells after 72 h incubation. Results were expressed as mean ± S.D. (n = 6) (**, *p*<0.05 *vs* docetaxel injection. *, *p*<0.05 *vs* DTX-SA-BSPs copolymer micelles)

The viability of Hela and HepG2 cells treated with blank SA-BSPs copolymer micelles, docetaxel injection and DTX-SA-BSPs copolymer micelles (with equal doses of DTX) for 72 h is shown in Fig 6. When drug concentration was 0.05 μg/mL, the cell viability of docetaxel injection and DTX-SA-BSPs copolymer micelles on HeLa cells were 60.3 ± 4.6% and 50.3 ± 3.9%, respectively, whereas at 0.5 μg/mL DTX concentration the cell viability was 55.5 ± 2.6% and 45.5 ± 1.9%, respectively (Fig 6A). The HepG2 cells viability was approximately 79.5 ± 3.4% and 46.5 ± 3.5% when treated with docetaxel injection, and 48.6 ± 0.4% and 16.5 ± 0.6% when treated with DTX-SA-BSPs copolymer micelles respectively, at a DTX concentration of 0.05 and 0.5 μg/mL (Fig 6B). A 50% increase in the growth inhibition was observed for HepG2 cells treated with DTX-SA-BSPs copolymer micelles compared to the cells treated with docetaxel injection after 72 h.

#### *In vivo* study

**Fig 7:**
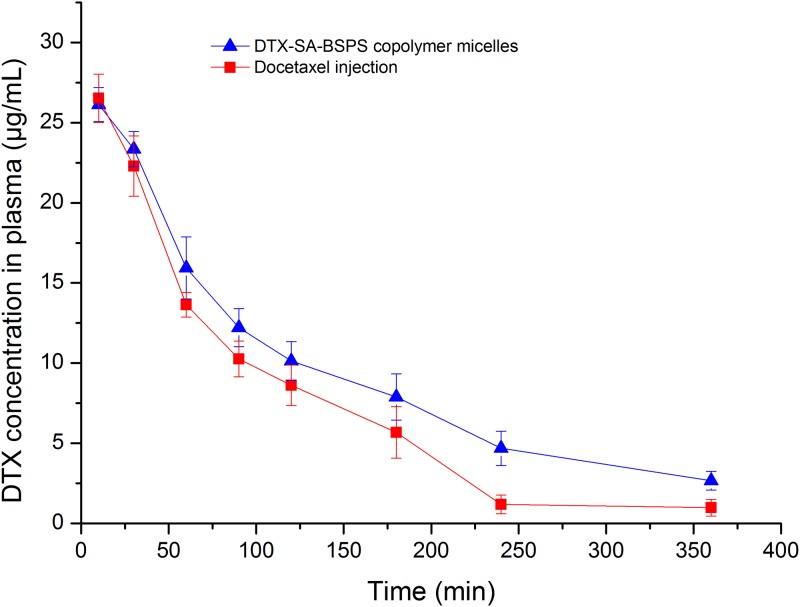
Mean plasma concentration-time curves of DTX in rats after a single *i*.*v*. dose (20 mg/kg) of DTX-SA-BSPs copolymer micelles and docetaxel injection *in vivo* pharmacokinetics study. Data were represented as mean ± S.D. of 3–5 experiments.

The mean plasma concentration-time profile of docetaxel injection and DTX-SA-BSPs copolymer micelles, injected intravenously, is shown in Fig 7, and the corresponding pharmacokinetic parameters are listed in Table 3. Several pharmacokinetic parameters, including clearance (CL), area of concentration-time curve (AUC_0-∞_), AUC_0-t_, and mean residence time (MRT) were shown to be different for DTX between docetaxel injection and DTX-SA-BSPs copolymer micelles. In case of DTX-SA-BSPs copolymer micelles, the AUC_0-∞_ and AUC_0-t_ values were significantly higher, with a little decrease in clearance (*p*<0.05) compared to the docetaxel injection. Further, the MRT was also extended (*p*<0.05) in DTX-SA-BSPs copolymer micelles. The AUC_0-∞_ of DTX-SA-BSPs copolymer micelles was approximately 1.37-fold higher than that of docetaxel injection (65.39±5.21 μg/mL h *vs* 47.73±0.49 μg/mL h, *p*<0.05).

**Table 3:** Plasma pharmacokinetic parameters of DTX-SA-BSPs copolymer micelles and docetaxel injection at a dose of 20 mg/kg of DTX after *i*.*v*. (data represented as mean±S.D.).

Parameters	Docetaxel injection	DTX-SA-BSPs copolymer micelles
t_1/2_ (h)	1.24±0.11	2.07±0.35*
MRT (h)	1.42±0.02	1.82±0.11*
CL (L/h/Kg)	0.42±0.03	0.31±0.02*
AUC_0-6h_ (mg/L h)	45.89±1.25	57.51±3.99*
AUC_0-∞h_ (mg/L h)	47.73±0.49	65.39±5.21*

*Significantly different from docetaxel injection (*p*<0.05) by Student *t*-test.

## Discussion

A new peak showing the characteristic absorption band at 1730 cm^-1^ in SA-BSPs was assigned to (-OCO-) group, further demonstrating successful conjugation of SA with BSPs. The characteristic absorption peak at 825.48 cm^-1^ showed the existence of mannose in BSPs [32]. Thus, the FTIR and ^1^H NMR techniques demonstrated that SA was successfully conjugated with BSPs.

The structural changes upon dilution of the SA-BSPs copolymer micelles solution in water was determined by fluorescence spectrophotometer with pyrene as a hydrophobic fluorescence probe. Pyrene is strongly emitted in hydrophobic condition or in a nonpolar environment, whereas, it is fairly quenched in polar solvent. Therefore, we investigated the self-aggregated behaviors of SA-BSPs copolymer micelles in water by using fluorescence excitation spectra. The CAC of SA-BSPs copolymer micelles was about 3.09 μg/mL, which is similar to the system of amphiphilic block copolymers [33]. At low concentration (concentration<CAC), there was negligible change in the fluorescence intensity, whereas, a remarkable increase was observe red in the intensity with increasing concentration, as shown by a previous report [26].

Amphiphilic copolymers, such as hydrophilic polysaccharides and block copolymers, have been reported to easily form nanosized carrier with a core-shell structure in an aqueous medium [34, 35]. These properties of amphiphilic block copolymers make them superior vehicles for entrapping and loading hydrophobic antitumor drugs [34]. SA-BSPs amphiphilic copolymers were synthesized through covalent attachment of SA to BSPs. The copolymer can easily self-assemble into micelles due to SA block in aqueous solution while BSPs can’t. The particle size of DTX-SA-BSP copolymer micelles 97.01 ± 3.17 nm was larger than that of blank SA-BSP copolymer micelles 60.52 ± 3.34 nm, indicating that particle diameter enlarged with DTX addition because DTX was carried to enter the hydrophobic cores of SA-BSPs copolymer micelles and resulted in the increase in volume of DTX-SA-BSPs copolymer micelles. The small size of the particles (less than 100 nm in diameter) has been shown to facilitate easy lymphatic transport and enhanced blood transmission of an antitumor drug, avoiding the reticuloendothelial system (RES) and passively delivering the antitumor drug [36].

The mean particle diameter and LC % showed an increasing trend, whereas, no distinct *zeta* potential change was observed, after loading the DTX below a 1:8 mass ratio of drug *verse* SA-BSPs copolymer. Therefore, we selected the mass ratio of 1:9 (drug/carrier) as the optimal formulation for further study, which was decided based on triplicate experiments. After lyophilization, the average EE and LC of freeze-dried DTX-SA-BSPs copolymer micelles were 8.97 ± 0.23% and 80.83 ± 0.49%, respectively. The results demonstrated that average LC and EE percentage decreased after freeze-dried process.

The release percentage of DTX from docetaxel injection was faster and higher than that from DTX-SA-BSPs copolymer micelles in the same aqueous media. The difference in DTX release rate was mainly because of the core-shell structure of SA-BSPs copolymer micelles. Lipophilic DTX was surrounded by the hydrophobic core-shell structure of the SA-BSPs, and the drug release was attributed to diffusion and dissolution [37]. These results revealed that DTX was gradually released from the DTX-SA-BSPs copolymer micelles, and a constant release rate was maintained for a relatively longer time. These properties of the micelles may reduce the injection frequency of the drug, which might be an encouraging strategy for their clinical application.

The viability of Hela and HepG2 cells treated with docetaxel injection was higher than that of cells treated with DTX-SA-BSPs copolymer micelles, at a drug concentration of 0.05 and 0.5 μg/mL. The results revealed that DTX-SA-BSPs copolymer micelles significantly decreased the cancer cell viability compared to docetaxel injection, perhaps because of better biocompatibility of SA-BSPs and DTX-SA-BSPs copolymer micelles. The DTX-SA-BSPs copolymer micelles were able to easily attach onto the cell surface, and accelerate the drug release near the cell membrane, thus developing a concentration gradient, further promoting the DTX penetration into the cell [38]. Carcinogenic cells possessing special endocytic activity internalized the BSPs graft copolymer micelles, which may have increased the drug concentration inside the cells. In addition, DTX-SA-BSPs copolymer micelles may have protected the DTX from the effect of P-glycoprotein (P-gp) pumps, which further resulted in increased drug concentration inside the cancerous cells. Moreover, intracellular delivery of DTX-SA-BSPs could have improved the drug concentration near the site of action [39].

The analysis of pharmacokinetic parameters between docetaxel injection and DTX-SA-BSPs revealed that DTX-SA-BSPs copolymer micelles were able to delay the elimination of DTX, and maintained a constant blood circulating concentration in rats. The increased AUC and elongated MRT of DTX-SA-BSPs copolymer micelles further indicated that the micelles might possess a longer blood circulating effect. The longer circulating effect may also be attributed to the location of DTX at the core of SA-BSPs copolymer micelles, whereas the hydrophilic shell can stabilize and protect the drug in the aqueous medium. The special core-shell structure delayed the degradation and slowed down the release of DTX compared to the docetaxel injection. The smaller size of DTX-SA-BSPs copolymer micelles (<200 nm) might help it in escaping from RES recognition [40], and could be another reason for being slowly removed from the circulation compared to the docetaxel injection.

## Conclusion

The present study was an effort to deliver DTX using nanoparticulate drug delivery system in order to minimize the toxicity associated with its use and improve its therapeutic efficacy. The SA-BSPs copolymer micelles were successfully applied as a macromolecular material to encapsulate the DTX using an emulsion method. The copolymer micelles displayed a high drug-loading capability and encapsulation efficiency with an average particle size of 97.01±3.17 nm. Compared to the docetaxel injection, the DTX-SA-BSPs copolymer micelles were more effective in inhibiting the growth of HepG2 and Hela cancer cells. The DTX-SA-BSPs copolymer micelles also maintained a constant release rate for a relatively longer time and stayed in plasma for longer than docetaxel injection. Absolute bioavailability of DTX-SA-BSPs copolymer micelles was 1.39-fold higher than that of docetaxel injection. Overall, DTX-SA-BSPs copolymer micelles might be an efficient way to increase the absolute bioavailability of poorly water-soluble drugs and an encouraging strategy for use in clinical application.
